# A Comprehensive Three-Dimensional Analysis of a Large-Scale Multi-Fuel CFB Boiler Burning Coal and Syngas. Part 1. The CFD Model of a Large-Scale Multi-Fuel CFB Combustion

**DOI:** 10.3390/e22090964

**Published:** 2020-08-31

**Authors:** Jaroslaw Krzywanski, Karol Sztekler, Mateusz Szubel, Tomasz Siwek, Wojciech Nowak, Łukasz Mika

**Affiliations:** 1 Faculty of Science and Technology, Jan Dlugosz University in Czestochowa, al.Armii Krajowej 13/15, 42-200 Czestochowa, Poland; 2 Faculty of Energy and Fuels, AGH University of Science and Technology, al. Mickiewicza 30, 30-059 Krakow, Poland; sztekler@agh.edu.pl (K.S.); mszubel@agh.edu.pl (M.S.); siwek@agh.edu.pl (T.S.); wnowak@agh.edu.pl (W.N.); lmika@agh.edu.pl (Ł.M.)

**Keywords:** circulating fluidized bed, CFD, multi-fuels boilers, modeling, simulation, co-combustion

## Abstract

The paper is focused on the idea of multi-fuel combustion in a large-scale circulating fluidized bed (CFB) boiler. The article discusses the concept of simultaneous coal and syngas combustion. A comprehensive three-dimensional computational fluid dynamics (CFD) model is developed, which allows us to describe complex phenomena that occur in the combustion chamber of the CFB boiler burning coal and syngas produced from coal sludge.

## 1. Introduction

Awareness of the growing environmental pollution and climate change is leading to the emergence of new methods to reduce emissions from fossil fuel combustion [1,2,3]. One of the most favored steam generation technology in recent times is circulating fluidized bed (CFB) technology. The main advantages of CFB boilers are stable operation, low acid gas emissions, and fuel flexibility with the potential for reliable operation with difficult-to-burn fuels [4,5,6].

Since the complexity of fuel combustion in the CFB boilers, especially in the large-scale units, is still not sufficiently recognized, the model research of such objects operation is of practical significance. Different modeling approaches to modeling CFB facilities can be distinguished, including artificial intelligence (AI) and the programmed computing approach [7,8,9,10]. The AI approach constitutes the use of the methods, which can reproduce a process from training samples [11,12,13,14]. The programmed computing approach base on writing algorithms to solve elaborate mathematical descriptions of the considered processes [5,6,15,16,17,18,19,20,21]. Basu et al. [5,6] provided a wide-range review and comparison of circulating fluidized bed combustor CFBC models. A similar qualification shows Gungor and Eskin et al. [22]. The authors selected three groups of CFB models, corresponding to three levels of sophistication. The Group I encompasses the 1D, mostly, plug flow/stirred tanks or axial solids distribution models, using simplified mass and energy balance. Group II contains 2D (1.5D), core/annulus models with a broad consideration of combustion and other related processes, Finally, Group III constitute 3D models, mainly CFD (computational fluid dynamics) models.

Models built using the CFD approach are the most general and numerically sophisticated, advanced, based on detailed consideration of chemical kinetics and individual physical processes, gas, and solids continuity equations, momentum balances, and appropriate constitutive equations [23]. Many various 3D models of CFB boilers exist in literature. 

An interesting application of a hybrid Euler–Lagrange approach to model the dense gas–solid flow combined with a combustion process in a large-scale industrial CFB boiler was developed in [24]. Modifications of the existing code via the user-defined function UDF mechanism as well as the selection of a proper combination of submodels, numerical techniques, and the careful control of the solution convergence implemented in the ANSYS Fluent allowed the successful validation of the developed model against the measured data sets [24].

A model for the simulation of reactive gas–solids flows in large, industrial CFB combustors is developed in [25]. The 105 MW_e_ Duisburg and the 235 MW_e_ Turów combustors are discussed at work. A semiempirical approach was employed to describe the 3D combustion of coal in [26]. The calculations were performed on the 12 MW_th_ CFB boiler of the Chalmers University of Technology is modeled. A three-dimensional numerical model has was successfully established to simulate olive cake combustion in a CFB combustor in [27]. The calculations were conducted for a laboratory-scale CFD model unit.

A comprehensive CFD combustion model for a large-scale supercritical CFB boiler was presented in [28]. The authors considered a large-scale 350 MW supercritical CFB boiler.

The fuel flexibility of CFB technology has caused a discussion on the feasibility of different fuels co-combustion in a CFB furnace. Thus the concept of multi-fuel CFB units has emerged. As a general rule, the kind of fuel dedicated to an individual boiler depends on various factors, including fuel properties and design parameters [29]. Since the CFB boilers allow using wide size distribution feedstock [30], many works are devoted to biomass and coal co-combustion [31,32,33], considering that biomass belongs to the environmentally friendly, renewable fuels. However, the authors underlined that the use of biofuels tends to operational problems during the combustion process due to, e.g., defluidization as the effect of the bed sintering, superheater fouling, and high-temperature corrosion. The main reason for such biofuels’ behavior is their high content of sodium and potassium, leading to lowering the ash softening point [4,15,34]. Two combustion approaches with wood-based biomass, coal, peat, and solid recovered fuel (SRF) utilization were discussed in [29]. The authors underlined the feasibility of using 100% SRF, and 100% demolition wood.

The co-combustion results of sewage sludge with coal and wood in laboratory-scale and pilot-scale 12 MW_th_ CFB boilers were considered in [35].

The interaction of fuels in co-firing in FBC was also thoroughly discussed by Huppa [36]. The author emphasized that some fuel mixtures show surprising interactions as there are no sufficient data about the behavior of the fuels when they are burned in the mixtures. Since the change in operational conditions occurs while the fuel type changes, it is difficult to draw out the process behavior. The author gives a review of some research on the fuel interaction in fluidized bed combustors. The cases with a gas co-combustion were also listed but not discussed [36].

The use of gaseous fuel, with a properly organized gas supply system, can be beneficial for power unit flexibility. A key issue is to provide the minimum technically justified load to avoid the instability of combustion [37]. However, an essential advantage of converting solid fuel into process gas is enabling stable operation of the boiler furnace with a substantial reduction of capacity. Additional positive aspects of the proposed concept are economic, social, and ecological benefits from the utilization of coal sludge [33,35,36].

On the other hand, the acceptable parameters may be exceeded, which may result in the destruction or, at best, reduction of the service life of individual components. Such effects may be a consequence of a change in the flue gas velocity and temperature profiles [4,18,37,38,39].

The above literature review reveals that the latest bibliography lacks such comprehensive 3D-CFD modeling of a large-scale, multi-fuel, CFB boiler, burning coal, and syngas.

In this paper, the multi-fuel coal and syngas combustion process in the large-scale OFz-425 CFB boiler installed in Poland was simulated. The commercial CFD ANSYS Workbench package, including the ANSYS Fluent Solver, was employed to describe flow and thermal conditions in the furnace of the CFB boiler. 

To the best of our knowledge, the present work is the first one in the open in the literature, such as a comprehensive 3D CFD model of a large-scale multi-fuel CFB boiler burning coal and syngas.

## 2. Description of the OFZ-425 CFB Boiler

**Figure 1:**
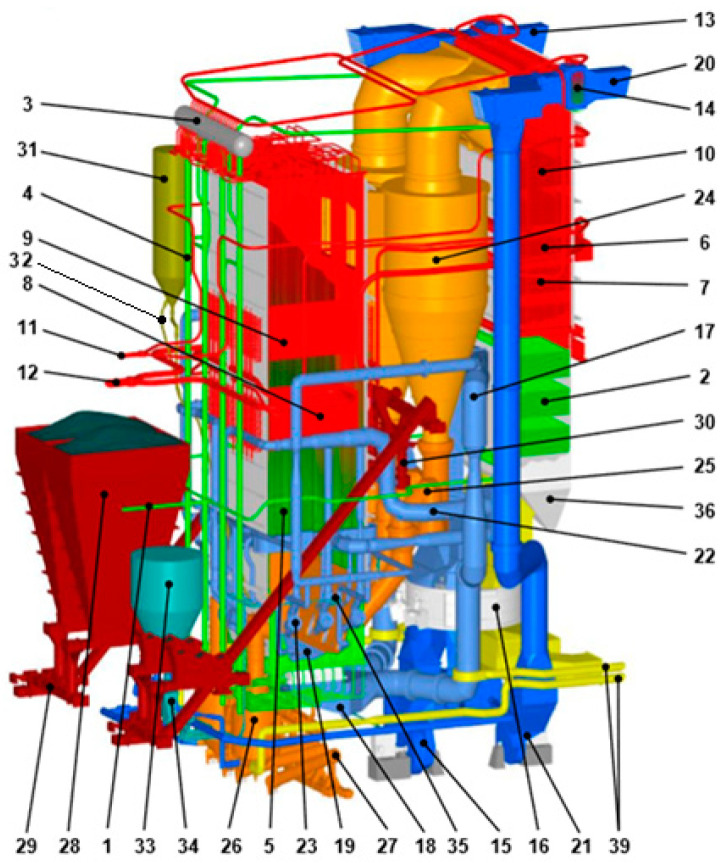
The OFz-425 circulating fluidized bed boiler, 1. Water inlet, 2. Economizer, 3. Drum, 4. Downpipes, 5. Combustion chamber, 6. Superheater I (SH I), 7. Reheater I (RH I), 8. Superheater II (SH II), 9. Reheater II (RH II), 10. Superheater III (SH III), 11. Fresh steam collector, 12. Secondary steam collector, 13. Primary air, 14. Steam air heater, 15. Air fan, 16. Air heater, 17. Primary air duct, 18. Wind box, 19. Air distributor, 20. Secondary air inlet, 21. Secondary air fan, 22. Secondary air ducts, 23. Start-up burners, 24. Cyclone, 25. Ash seal, 26. Ash separator, 27. Bottom ash feeder, 28. Coal hopper, 29, 30. Coal Feeders, 31. Inert material tank, 32. Installation of preparation and feeding of inert material, 33. Limestone hopper, 34. Limestone feeding installation, 35. Limestone feeding nozzles, 36. Second flue gas duct.

The simulations are conducted for the OFz-425 large-scale CFB boiler, produced by RAFAKO S.A., Poland. It is a two-pass CFB unit burning bituminous coal combustion (Figure 1). The main parts of the boiler are the riser with the combustion chamber made of membrane-walls, superheater II (SH II), and reheater II (RH II), the flue gas discharge with cyclones, and the second pass in the convection cage consisting of built-in superheaters and reheaters as well as an economizer and a rotary air heater. 

The technical data of the boiler are listed in Table 1.

Bituminous coal is the primary fuel dedicated to the CFB boiler. Its properties are listed in Table 2.

One of the options for processing coal sludge is its gasification in gasification reactors and then the use of the obtained syngas as an auxiliary fuel in power boilers [2,40]. The feasibility of coal and syngas co-combustion in the OFz-425 boiler is considered during the study. One of the syngas production technologies is the use of gasification technology proposed by Synthesis Energy Systems, Inc. (SES), Huston USA. During the gasification mixture of 70% coal sludge (8% moisture) and 30% fine coal, the gas product with the properties given in Table 3 is obtained.

The amount of batch sand is equal to 64,500 kg, of which 50,000 kg fills the furnace chamber, and the rest is contained in the seals of the boiler. 

## 3. Methods

The defined research problem concerns the analysis of CFD modeling of the multi-fuel OFz-425 CFB boiler supplied by bituminous coal and syngas from coal sludge. The purpose of the model research is to recognize thermal-flow conditions in the CFB boiler in a nominal operating configuration and under the modified mode, i.e., with the supply of the auxiliary syngas. 

ANSYS software was used during the model research, as the world’s leading simulation package, enabling comprehensive calculations in almost every field of science and industry. The mathematical model of the OFz 425 CFB boiler base on the algorithms of numerical flow simulations with chemical reactions that occur in the furnace. The two-phase model of homogenous and heterogeneous combustion employs the computational mechanics of CFD fluids and transport equations for the flow of the reactive mixture. The equations for mass, enthalpy, momentum, and selected gaseous components relevant to the combustion process are applied using the finite volume method.

The practical purpose of the study is to determine the increase in temperatures inside the combustion chamber as a result of the syngas supply and to compare the results with the critical operating temperatures of the fluidized bed. The mass balance and solid-phase transport DPM (discrete phase modeling) equations are considered in the model.

The Eddy dissipation model is employed with the radiation heat transfer equations, according to the discrete ordinates model. 

In order to accurately simulate flow phenomena in non-laminar flows, Reynolds averaged Navier–Stokes equations (RANS) are supplemented with the k-omega BSL turbulence model, based on the Boussinesq hypothesis. 

The equations are solved in the ANSYS Fluent solver based on the finite volume method. A detailed description of the physical models used in the paper and the methods of numerical solutions of the equations are described in the consecutive subsections.

### 3.1. Transport Equations with Combustion (Eddy Dissipation Method)

The mass conservation equation (the continuity equation) can be written as (1):

(1) $$\frac{\partial \rho }{\partial t}+\nabla \cdot \left(\rho \bar{v}\right)=S_{m}$$

Equation (1) is a general form of mass conservation equation for both compressible and incompressible flows, where the S_m_ is the mass source. 

Equation (1) for 2D axial-symmetric issues can be written, as follows:

(2) $$\frac{\partial \rho }{\partial t}+\frac{\partial }{\partial x}\left({\rho v}_{x}\right)+\frac{\partial }{\partial r}\left({\rho v}_{r}\right)+\frac{{\rho v}_{r}}{r}=S_{m}$$

In an inertial system (without acceleration), the momentum conservation equation can be described by the Equation (3):

(3) $$\frac{\partial }{\partial t}\left(\rho \bar{v}\right)+\nabla \cdot \left(\rho \bar{v}\bar{v}\right)=-\nabla p+\nabla \cdot \left(\bar{\bar{\tau }}\right)+\rho \bar{g}+\bar{F}$$

where: p is static pressure, $\bar{\bar{\tau }}$ is the stress tensor (described below), $\rho \bar{g}$ is the gravity, and $\bar{F}$ expresses the external mass forces and other source terms present in the model.

The stress tensor mentioned above ($\bar{\bar{\tau }}$) is defined as (4):

(4) $$\bar{\bar{\tau }}=\mu \left[\left(\nabla \bar{v}+\nabla {\bar{v}}^{T}\right)-\frac{2}{3}\nabla \bar{v}I\right]$$

where μ is the dynamic viscosity, and I stands for the unit tensor.

Three mechanisms of heat transport, i.e., conduction, convection, and radiation, can be considered in the energy equation of the following form (5):

(5) $$\frac{\partial }{\partial t}\left(\rho E\right)+\nabla \cdot \left(\bar{v}\left(\rho E+p\right)\right)=\nabla \cdot \left(k_{\mathrm{eff}}\nabla T-\underset{j}{\sum }h_{j}{\bar{J}}_{j}+\left({\bar{\bar{\tau }}}_{\mathrm{eff}}\cdot \bar{v}\right)\right)+S_{h}$$

where $k_{\mathrm{eff}}$ is an effective conduction coefficient, ${\bar{J}}_{j}$ the mass flow rate of a diffusive component j, the next terms of Equation (5) define energy transfer by conduction, diffusion of substances, viscous dissipation, and the term $S_{h}$ is a user-defined term, representing volume heat source.

E from Equation (5) is defined as (6):

(6) $$E=h-\frac{p}{\rho }+\frac{v^{2}}{2}$$

where h-enthalpy is expressed for ideal gases as:

(7) $$h=\underset{j}{\sum }Y_{j}h_{j}$$

and for incompressible flows (8):

(8) $$h=\underset{j}{\sum }Y_{j}h_{j}+\frac{p}{\rho }$$

The term $Y_{j}$ in Equations (7) and (8) stands for the mass fraction of component j and $h_{j}$ is expressed by Equation (9):

(9) $$h_{j}=\overset{T}{\underset{T_{\mathrm{ref}}}{\int }}c_{p,j}\;\mathrm{dT}$$

The reference temperature $T_{\mathrm{ref}}$, employed to calculate the enthalpy, depends on the solver and the models used.

In the combustion model, without premixing, the energy equation in the form of the total enthalpy Equation (10) can be written:

(10) $$\frac{\partial }{\partial t}\left(\rho H\right)+\nabla \cdot \left(\rho \bar{v}H\right)=\nabla \cdot \left(\frac{k_{\mathrm{eff}}}{C_{p}}\nabla H\right)+S_{h}$$

Assuming that the Lewis number (Le) is equal to 1, the conduction and the diffusion term of the substance combine forming the first term in Equation (10), and the viscous dissipation is included in the second term of the above equation. Total H is defined as follows:

(11) $$H=\underset{j}{\sum }Y_{j}H_{j}$$

where $Y_{j}$ is the mass fraction of the component j and

(12) $$H_{j}=\overset{T}{\underset{T_{\mathrm{ref},j}}{\int }}c_{p,j}\mathrm{dT}+h_{j}^{0}\left(T_{\mathrm{ref},j}\right)$$

where $h_{j}^{0}\left(T_{\mathrm{ref},j}\right)$ is the enthalpy of the substance i formation at the reference temperature.

When heat is supplied to a fluid, its density changes with temperature, generating convection. The effect of buoyancy forces on mixed convection can be determined by the ratio of Grashof and Reynolds numbers (3.13):

(13) GrRe2=gβΔTLν2

For the pure form of natural convection, the importance of buoyancy-induced flow can be determined by the Rayleigh number (14):

(14) $$\mathrm{Ra}=\frac{{g\beta \Delta \mathrm{TL}}^{3}\rho }{\mu \alpha }$$

where: β is the thermal expansion coefficient (15):

(15) $$\beta =-\frac{1}{\rho }{\left(\frac{\partial \rho }{\partial T}\right)}_{p}$$

The value of the thermal diffusivity factor is expressed as (16):

(16) $$\alpha =\frac{k}{{\rho C}_{p}}$$

When modeling of combustion processes, the transport and mixing components, as well as chemical reactions, should also be considered. For this purpose, ANSYS Fluent allows introducing introduces an additional Equation (17):

(17) $$\frac{\partial }{\partial t}\left({\rho Y}_{i}\right)+\nabla \cdot \left({\rho \;\bar{v}Y}_{i}\right)=-\nabla \cdot {\bar{J}}_{i}+R_{i}+S_{i}$$

where: $R_{i}$ is the index of net production of the substance i as a result of chemical reactions, and $S_{i}$ means the source term. Equation (17) is solved for N − 1 substances, where N is the total number of fluid phase substances present in the system.

The term $R_{i}$ expressing the formation rate of the substance i via chemical reactions for turbulent flows can be calculated using the Eddy dissipation model. This model of chemical–turbulent interaction assumes that the reaction rate is controlled by turbulence ignoring chemical time scales. Such an approach allows avoiding calculating the chemical kinetics of Arrhenius, which computationally are very demanding [41]. 

On the basis of this model, the production rate $R_{i,r}$ of a substance i as a result of the chemical reaction r. The $R_{i,r}$ is the lower value of the following two (18) and (19):

(18) $$R_{i,r}=\nu_{i,r}^{\prime }M_{w,i}A\rho \frac{\varepsilon }{k}\min_{ℛ}\left(\frac{Y_{ℛ}}{\nu_{ℛ,r}^{\prime }M_{w,ℛ}}\right)$$

(19) $$R_{i,r}=\nu_{i,r}^{\prime }M_{w,i}\mathrm{AB}\rho \frac{\varepsilon }{k}\left(\frac{\sum_{P}Y_{P}}{\sum_{j}^{N}\nu_{j,r}^{\prime \prime }M_{w,j}}\right)$$

where: $Y_{P}$—the mass fraction of the product, P, $Y_{ℛ}$—the mass fraction of a considered reagent, ℛ, A—an empirical constant of 4.0, and B—an empirical constant of 0.5.

### 3.2. K-Omega BSL Turbulence Model

For an average turbulent fluid movement, each flow parameter at a given time can be expressed as the sum of the averaged value and its fluctuation. For a given velocity component, we can write:

(20) $$u_{i}=\bar{u_{i}}+u_{i}^{\prime }$$

In the same way, we may write scalars:

(21) $$\phi =\bar{\phi }+\phi^{\prime }$$

where ϕ indicates a scalar, such as pressure, energy, or concentration. 

The following form of transport equations in the tensor notation can be formulated:

(22) $$\frac{\partial \rho }{\partial t}+\frac{\partial }{\partial x_{i}}\left({\rho u}_{i}\right)=0$$

(23) $$\frac{\partial }{\partial t}\left({\rho u}_{i}\right)+\frac{\partial }{\partial x_{j}}\left({\rho u}_{i}u_{j}\right)=-\frac{\partial p}{\partial x_{i}}+\frac{\partial }{\partial x_{j}}\left[\mu \left(\frac{\partial u_{j}}{\partial x_{j}}+\frac{\partial u_{j}}{\partial x_{i}}-\frac{2}{3}\delta_{\mathrm{ij}}\frac{\partial u_{j}}{\partial x_{j}}\right)\right]+\frac{\partial R_{\mathrm{ij}}}{\partial x_{j}}$$

where R_ij_ is a tenor of Reynolds’ stress, related to the average rate of momentum change due to turbulent pulsations. In the Cartesian coordinate system, this tenor has a form consistent with Equation (24):

(24) $$R_{\mathrm{ij}}=-\rho \bar{u_{j}^{\prime }}\bar{u_{i}^{\prime }}=\left[\begin{array}{ccc} -\rho \bar{{\left(u_{x}^{\prime }\right)}^{2}} & -\rho \bar{u_{x}^{\prime }}\bar{u_{y}^{\prime }} & -\rho \bar{u_{x}^{\prime }}\bar{u_{z}^{\prime }}\\ -\rho \bar{u_{y}^{\prime }}\bar{u_{x}^{\prime }} & -\rho \bar{{\left(u_{y}^{\prime }\right)}^{2}} & -\rho \bar{u_{y}^{\prime }}\bar{u_{z}^{\prime }}\\ -\rho \bar{u_{z}^{\prime }}\bar{u_{x}^{\prime }} & -\rho \bar{u_{j}^{\prime }}\bar{u_{i}^{\prime }} & -\rho \bar{{\left(u_{z}^{\prime }\right)}^{2}} \end{array}\right]$$

The problem of missing six equations for each component in the Reynolds tensor can be solved by solving the influence of turbulence, according to the Boussinesq approach [42]:

(25) $$R_{\mathrm{ij}}=-\rho \bar{u_{j}^{\prime }}\bar{u_{i}^{\prime }}=\mu_{T}\left(\frac{\partial {\bar{u}}_{i}}{\partial x_{j}}+\frac{\partial {\bar{u}}_{j}}{\partial x_{i}}\right)-\frac{2}{3}\left(\rho k+\mu_{T}\frac{\partial u_{k}}{\partial x_{k}}\right)\delta_{\mathrm{ij}}$$

where $\mu_{T}$ stands for turbulent viscosity. It is a scalar function (often non-linear) of several variables, such as the physicochemical properties of the fluid or the nature of the flow. 

The baseline turbulence (BSL) model k-ω was employed in the paper as it combines in a hybrid way the advantages of the k-ε model for free flow and the k-ω model solved in the wall area. The balance equations for the individual components of the model takes the following form:

(26) $$\frac{\partial }{\partial t}\left(\rho k\right)+\frac{\partial }{\partial x_{i}}\left({\rho \mathrm{ku}}_{i}\right)=\frac{\partial }{\partial x_{j}}\left(\Gamma_{k}\frac{\partial k}{\partial x_{j}}\right)+G_{k}-Y_{k}+S_{k}$$

(27) $$\frac{\partial }{\partial t}\left(\rho \omega \right)+\frac{\partial }{\partial x_{i}}\left({\rho \omega u}_{i}\right)=\frac{\partial }{\partial x_{j}}\left(\Gamma_{\omega }\frac{\partial \omega }{\partial x_{j}}\right)+G_{\omega }-Y_{\omega }+D_{\omega }+S_{\omega }$$

where $G_{k}$ expresses the kinetic energy turbulence production and is defined in the same way as in the standard model k-ω and $G_{\omega }$ represents the production of ω. Terms $\Gamma_{k}$ and $\Gamma_{\omega }$ are the effective diffusion coefficients of the k and ω variables, while $Y_{k}$ and $Y_{\omega }$ are the terms of the variable dissipation of the k and ω due to turbulence, $D_{\omega }$ is the cross-diffusion term, while $S_{k}$ and $S_{\omega }$ stand for user-defined function sources.

The above mentioned effective diffusion coefficients are determined by the following Equations (28) and (29):

(28) $$\Gamma_{k}=\mu +\frac{\mu_{t}}{\sigma_{k}}$$

(29) $$\Gamma_{\omega }=\mu +\frac{\mu_{t}}{\sigma_{\omega }}$$

where $\sigma_{k}$ and $\sigma_{\omega }$ are mean turbulent Prandtl’s numbers for the k and ω variables, defined by (30) and (31):

(30) $$\sigma_{k}=\frac{1}{\frac{F_{1}}{\sigma_{k,1}}+\frac{\left(1-F_{1}\right)}{\sigma_{k,2}}}$$

(31) $$\sigma_{\omega }=\frac{1}{\frac{F_{1}}{\sigma_{\omega ,1}}+\frac{\left(1-F_{1}\right)}{\sigma_{\omega ,2}}}$$

Turbulent viscosity $\mu_{t}$ is expressed by the Equation (32):

(32) $$\mu_{t}=\alpha^{*}\frac{\rho k}{\omega }$$

where $\alpha^{*}$ stands the correction factor for low Reynolds numbers given by Equation (33):

(33) α*=α∞*(α0*+Ret/Rk1+Ret/Rk)

and Ret=ρkμω, $R_{k}=6$, $\alpha_{0}^{*}=\frac{\beta_{i}}{3}$, and $\beta_{i}=0.072$.

The mixing function $F_{1}$ is defined by the Equation (34):

(34) $$F_{1}=\tan h\left(\Phi_{1}^{4}\right)$$

where: $\Phi_{1}=\min\left[\max\left(\frac{\sqrt{k}}{0.09\omega y},\frac{500\mu }{{\rho y}^{2}\omega }\right),\frac{4\rho k}{\sigma_{\omega ,2}D_{\omega }^{+}y^{2}}\right]$, $D_{\omega }^{+}=\max\left[2\rho \frac{1}{\sigma_{\omega ,2}}\frac{1}{\omega }\frac{\partial k}{\partial x_{j}}\frac{\partial \omega }{\partial x_{j}},10^{-10}\right],$ and y is the distance to the next surface.

The term $G_{k}$ in Equation (26) is defined by Equation (35):

(35) $$G_{k}=-\rho \bar{u_{i}^{\prime }u_{j}^{\prime }}\frac{\partial u_{j}}{\partial x_{i}}$$

and the term $G_{\omega }$ in Equation (27) describes the Equation (36):

(36) $$G_{\omega }=\frac{{\alpha \alpha }^{*}}{\nu_{t}}G_{k}$$

The dissipation of kinetic energy of turbulence $Y_{k}$ is defined similarly to the standard model k−ω and determined by Equation (37):

(37) $$Y_{k}={\rho \beta }^{*}k\omega$$

For the k-omega BSL model the term $f_{\beta^{*}}$, is equal to 1.

The dissipation of the ω variable is defined in a similar way as in the standard model. The difference constitutes in the way how $f_{\beta }$ and $\beta_{i}$ are calculated. In the model k-omega BSL, the value of $f_{\beta }$ is constant and equals 1, so $Y_{\omega }$ is ultimately expressed by Equation (38):

(38) $$Y_{k}={\rho \beta \omega }^{2}$$

where $\beta_{i}$ is defined by as follows:

(39) $$\beta_{i}=F_{1}\beta_{i,1}+\left(1-F_{1}\right)\beta_{i,2}$$

The term of the cross-diffusion $D_{\omega }$ was formed by combining the equations of the model k−ε and k−ω. Finally, this quantity is expressed by Equation (40):

(40) $$D_{\omega }=2\left(1-F_{1}\right)\rho \frac{1}{{\omega \sigma }_{\omega ,2}}\frac{\partial k}{\partial x_{j}}\frac{\partial \omega }{\partial x_{j}}$$

There are many constants in the model equations, which are listed below: $\sigma_{k,1}=2.0$, $\sigma_{\omega ,1}=2.0,$ $\sigma_{k,2}=1.0,$ $\sigma_{\omega ,2}=1.168,$ $\beta_{i,1}=0.075,$ and $\beta_{i,2}=0.0828$. Additional constants used in the equations of the k-omega BSL model take the same values as for the standard model k−ω.

### 3.3. Discrete Ordinates Radiation Model 

A radiation model solving the radiation transport Equation (41) for a finite number of discrete solid angles was introduced into the CFD fluidized bed model. Radiative transfer equation (RTE) for the absorption, emitting, and dispersing medium in the position $\bar{r}$ and in the direction $\bar{s}$ has the following form:

(41) $$\frac{\mathrm{dI}\left(\bar{r},\;\bar{s}\right)}{\mathrm{ds}}+\left(a+\sigma_{s}\right)I\left(\bar{r},\;\bar{s}\right)={\mathrm{an}}^{2}\frac{{\sigma T}^{4}}{\prod }+\frac{\sigma_{s}}{4\prod }\overset{4\prod }{\underset{0}{\int }}I\left(\bar{r},{\;\bar{s}}^{\prime }\right)\Phi \left(\bar{s}\cdot {\bar{s}}^{\prime }\right)d\;\Omega^{\prime }$$

where: $\bar{r}$—location vector, $\bar{s}$—direction vector, ${\bar{s}}^{\prime }$—vector of scattering direction, s—path length, a—absorption coefficient, n—refraction index, $\sigma_{s}$—refraction index, σ—Stefan-Boltzmann’s constant, I—radiation intensity depending on location ($\bar{r}$) and direction ($\bar{s}$), T—local temperature, and $\Omega^{\prime }$—solid angle. Equation (41) is transformed into a transport equation for radiation intensity in the spatial coordinate system (x,y,z). 

The discrete ordinates (DO) model uses the radiation transport equation in the direction $\bar{s}$ as a surface equation:

(42) $$\nabla \left(I\left(\bar{r},\;\bar{s}\right)\;\bar{s}\right)+\left(a+\sigma_{s}\right)I\left(\bar{r},\;\bar{s}\right)={\mathrm{an}}^{2}\frac{{\sigma T}^{4}}{\prod }+\frac{\sigma_{s}}{4\prod }\overset{4\prod }{\underset{0}{\int }}I\left(\bar{r},{\;\bar{s}}^{\prime }\right)\Phi \left(\bar{s}\cdot {s¯}^{\prime }\right)d\Omega^{\prime }$$

It is also possible to consider non-grey radiation using a grey-band model. In this case, Equation (42) takes the Equation (43):

(43) $$\nabla \left(I_{\lambda }\left(\bar{r},\;\bar{s}\right)\;\bar{s}\right)+\left(a_{\lambda }+\sigma_{s}\right)I_{\lambda }\left(\bar{r},\;\bar{s}\right)=a_{\lambda }I_{b\lambda }+\frac{\sigma_{s}}{4\prod }\overset{4\prod }{\underset{0}{\int }}I_{\lambda }\left(\bar{r},{\;\bar{s}}^{\prime }\right)\Phi \left(\bar{s}\cdot {\bar{s}}^{\prime }\right)d\Omega^{\prime }$$

In the above equation, λ is the wavelength, $a_{\lambda }$ is the absorption coefficient and $I_{b\lambda }$ states for the radiation intensity of the black body, determined by Planck’s function. 

Such an implementation of the DO model divides the radiation spectrum into N bands with different wavelength ranges. These bands do not have to be adjacent or equal. The user enters the wavelength ranges in the individual bands himself. The RTE equation is then integrated into each defined band, leading to transport equations of $I_{\lambda }$ Δλ, the radiation energy in each wavelength range Δλ. Then the radiation in each band is considered to be grey.

The emissivity of a black body in a given range of wavelengths per unit solid angle is defined as (44):

(44) $$\left[F\left(0\to {n\lambda }_{2}T\right)-F\left(0\to {n\lambda }_{1}T\right)\right]n^{2}\frac{{\sigma T}^{4}}{\prod }$$

where F(0→nλT) stands for a portion of the radiation energy emitted by the black body.

The total radiation intensity $I\left(\bar{r},\;\bar{s}\right)$ in each direction $\bar{s}$ in the position $\bar{r}$ is calculated by summing up all wavelength ranges according to the Equation (45):

(45) $$I\left(\bar{r},\;\bar{s}\right)=\underset{k}{\sum }I_{\lambda_{k}}\left(\bar{r},\;\bar{s}\right){\Delta \lambda }_{k}$$

The radiation DO model in the considered case is complemented by the weighted sum of gray gases model (WSGGM) to calculate the variable value of the absorption coefficient. 

The model makes a compromise between the excessive simplification of the gray gas model and the complete model considering absorption in individual bands.

The WSGGM model, based on the assumption that total emissivity on the s path, can be expressed as:

(46) $$\varepsilon =\overset{I}{\underset{i=0}{\sum }}a_{\varepsilon ,i}\left(T\right)\left(1-e^{-\kappa_{i}\mathrm{ps}}\right)$$

where $a_{\varepsilon ,i}$ stands for the emissivity factor for the fictitious grey gas i, the value in brackets is the emissivity of the fictitious grey gas i and $\kappa_{i}$ is the absorption coefficient of the grey gas i. The value p is the sum of the partial pressures of all the absorbing component gases.

Terms $a_{\varepsilon ,i}$ and $\kappa_{i}$ are obtained according to [43,44] and depend on the gas composition, but the coefficient $a_{\varepsilon ,i}$ is also a function of temperature. If the total pressure is not equal to 1 atm, the scaling principle for the coefficient $\kappa_{i}$ is applied.

For $p_{\mathrm{tot}}<0.9\;\mathrm{atm}$ or $p_{\mathrm{tot}}>1.1\;\mathrm{atm}$ a correction is introduced, according to the expression:

(47) $$\kappa_{i}\to \kappa_{i}p_{\mathrm{tot}}{}^{m}$$

where: m is determined according to [45] and depends on partial pressures and temperatures of the absorbing gas components.

The absorption coefficient for i=0 is equal to 0 to include windows in the radiation spectrum between highly absorbent regions $\left(\sum_{i=1}^{I}a_{\varepsilon ,i}<1\right)$, whereas the weight for i=0 is determined from the Equation (48):

(48) $$a_{\varepsilon ,0}=1-\overset{I}{\underset{i=1}{\sum }}a_{\varepsilon ,i}$$

The influence of temperature on the coefficient $a_{\varepsilon ,i}$ is usually approximated using the following formula:

(49) $$a_{\varepsilon ,i}=\overset{J}{\underset{j=1}{\sum }}b_{\varepsilon ,i,j}T^{j-1}$$

where $b_{\varepsilon ,i,j}$ stands for polynomial coefficients of temperature emissivity of gases, while $b_{\varepsilon ,i,j}$ and $\kappa_{i}$ are estimated by matching Equation (46) to the total emissivity obtained experimentally [43,44,46].

The coefficients $b_{\varepsilon ,i,j}$ and $\kappa_{i}$ can be considered as constant, as they change slightly with ps and T, according to [43,44]. If $\kappa_{i}\mathrm{ps}\;“1$ for all values i Equation (46) can be simplified as follows (50):

(50) $$\varepsilon =\overset{I}{\underset{i=0}{\sum }}a_{\varepsilon ,i}\kappa_{i}p$$

When comparing Equation (50) with the grey gas model with absorption coefficient α, it is observed that the radiation intensity change over distance s in the WSGGM model is the same as in the grey gas model with α:

(51) $$\alpha =\overset{I}{\underset{i=0}{\sum }}a_{\varepsilon ,i}\kappa_{i}p$$

In the general case α is calculated as (52):

(52) $$\alpha =-\frac{\ln\left(1-\varepsilon \right)}{s}$$

where the emissivity ε for the WSGGM model is calculated from the Equation (36).

The factor α defined by Equation (52) is dependent on s, which reflects the non-grey nature of radiation absorption in molecular gases. ANSYS Fluent employs the Equation (51) for $s\leq 10^{-4}\;m$ and the Equation (52) for $s>10^{-4}\;m$. For the border value s, both equations lead to an almost identical value.

### 3.4. Discrete Phase Modeling (DPM)

Currently, two approaches to numerical modeling of multiphase flows are most commonly used: Euler–Lagrange or Euler–Euler method. In the fluidized bed model, the Euler–Lagrange method was adapted. In this method, the fluid phase is treated as continuous by solving Navier–Stokes equations, while the dispersed phase is calculated by tracking a large number of particles, bubbles or droplets in the computing domain. The dispersed phase can exchange momentum, mass, and energy with a continuous fluid phase.

This approach becomes much simpler when interactions between particles can be neglected. This assumption requires the dispersed phase to have a small volume share. However, a high flow load of the dispersed phase (${\dot{m}}_{\mathrm{particles}}\geq {\dot{m}}_{\mathrm{fluid}}$) is also acceptable. Particle or droplet trajectories are calculated individually at specified intervals when performing fluid phase calculations making the approach suitable for modeling spray dryers, combustion of coal, and liquid fuels as well as two-phase flows. 

The force equation can be expressed by (53):

(53) $$\frac{{d\bar{u}}_{p}}{\mathrm{dt}}=\frac{\bar{u}-{\bar{u}}_{p}}{\tau_{r}}+\frac{\bar{g}\left(\rho_{p}-\rho \right)}{\rho_{p}}+\bar{F}$$

where $\bar{F}$ means a term of additional acceleration (force/unit of mass of the particle), the term $\frac{\bar{u}-{\;\bar{u}}_{p}}{\tau_{r}}$ stands for the drag force corresponding to the unit of mass of the particle. 

The particle relaxation time $\tau_{r}$ is defined as follows:

(54) τr=ρpdp218μ14CdRe

where $\bar{u}$ and ${\bar{u}}_{p}$ mean fluid and particles velocities, respectively, μ expresses the molecular viscosity, ρ and $\rho_{p}$ are fluid and particle density, respectively and $d_{p}$ stands for the particle diameter. The Re expresses Reynolds number:

(55) Re≡ρdp| u¯p− u¯|μ

Particle rotation has a significant impact on the trajectory of a given particle, moving in a fluid. An additional differential equation for the particle momentum (56) was employed to take into account this effect of particle rotation:

(56) $$I_{p}\frac{{d\bar{\omega }}_{p}}{\mathrm{dt}}=\frac{\rho_{f}}{2}{\left(\frac{d_{p}}{2}\right)}^{5}C_{w}\;\bar{\Omega }=\bar{T}$$

where $I_{p}$ is the moment of inertia, ${\bar{\omega }}_{p}$ expresses the angular velocity of the particle, $\rho_{f}$ is the density of the fluid, $d_{p}$ is the diameter of the particle, $C_{w}$ is the rotational resistance coefficient, $\bar{T}$ is the torque applied to the particle, and $\bar{\Omega }$ means the relative rotational speed between the particle and the fluid. The value $\bar{\Omega }$ is determined as (57):

(57) $$\bar{\Omega }=\frac{1}{2}\nabla {\;x\;\bar{u}}_{f}-{\bar{\omega }}_{p}$$

The moment of inertia for a spherical particle is calculated from Equation (58):

(58) $$I_{p}=\frac{\prod }{60}\rho_{f}d_{p}^{5}$$

Equation (53) contains the term $\bar{F}$, responsible for additional forces acting on the particle. They may be particularly relevant in specific circumstances. The first is the “virtual mass” force, which is the force needed to accelerate the fluid surrounding the particle. This force is determined by Equation (59):

(59) $$\bar{F}=C_{\mathrm{vm}}\frac{\rho }{\rho_{p}}\left({\bar{u}}_{p}\nabla \;\bar{u}-\frac{{d\;\bar{u}}_{p}}{\mathrm{dt}}\right)$$

where: $C_{\mathrm{vm}}$ stands for a virtual mass factor with a default value of 0.5.

This additional force is created by the pressure gradient (60):

(60) $$\bar{F}=\frac{\rho }{\rho_{p}}{\;\bar{u}}_{p}\nabla \bar{u}$$

Virtual mass and forces related to the pressure gradient are not relevant when the density of a fluid is significantly lower than the density of particles, as in the considered here case with solid particles in gaseous flows $\left(\frac{\rho }{\rho_{p}}\text{”}1\right)$. However, it is recommended to take them into account for density ratios higher than 0.1.

When particles are suspended in gas with large temperature gradients, an additional force acts on the particles in the opposite direction to this gradient. This phenomenon is referred to as thermophoresis (or otherwise thermodiffusion, Soret effect) and can be considered by an additional acceleration term in the Formula (53). It is expressed by Equation (61):

(61) $$\bar{F}=-D_{T,p}\frac{1}{m_{p}T}\nabla T$$

where $D_{T,p}$ expresses the thermophoresis thermoforming factor. This coefficient can be expressed as a constant, a polynomial, user-defined function, or can be expressed in the form proposed by Talbot [47], making that the Equation (61) takes the following form:

(62) $$\bar{F}=-\frac{6\prod d_{p}\mu^{2}C_{s}\left(K+C_{t}\mathrm{Kn}\right)}{\rho \left(1+3C_{m}\mathrm{Kn}\right)\left(1+2K+2C_{t}\mathrm{Kn}\right)}\frac{1}{m_{p}T}\nabla T$$

where Kn—Knudsen number $\left(\frac{2\lambda }{d_{p}}\right)$, λ—mean free fluid path, $K=k/k_{p}$, k—thermal conductivity of the fluid based on translation energy $\left(\frac{15}{4}\mu R\right)$, $k_{p}$—thermal conductivity of the particle, $C_{s}=1.17$, $C_{t}=2.18$, $C_{m}=1.14$, $m_{p}$—mass of the particle, T—local fluid temperature, and μ—fluid viscosity.

### 3.5. Finite Volume Method

For this work, the finite volume method was used. This method consists of dividing the examined area into a finite number of control cells with computational nodes. Then the differential equations describing a phenomenon are integrated into the control volume of each cell. Different types of approximation expressions replace individual parts of the equations. Finally, a system of algebraic equations, usually non-linear, is obtained [48].

The general form of the equation, which describes the transport of a variable in the differential form, is as follows [42]:

(63) $$\frac{\partial \left(\rho \phi \right)}{\partial t}+\mathrm{div}\left(\rho \phi \bar{u}\right)=\mathrm{div}\left(\Gamma_{\phi }\mathrm{grad}\phi \right)+S_{\phi }$$

The variable ϕ expresses a variable in the equations used to describe fluid flow. Replacing ϕ with an appropriate variable (u, v, and w) and selecting the appropriate diffusion coefficient, the individual transport equations described in Chapter 3.1 may be obtained.

The control volume method uses the general transport Formula (63). This equation is integrated over the entire volume of the control cell, and then transformed using the Ostrogradsky–Gauss theorem [42]:

(64) $$\overset{}{\underset{\mathrm{CV}}{\int }}\mathrm{diva}\mathrm{dV}=\overset{}{\underset{A}{\int }}n\cdot \mathrm{adA}$$

Finally, the following equation is obtained [42]:

(65) $$\frac{\partial }{\partial t}\left(\overset{}{\underset{\mathrm{CV}}{\int }}\left(\rho \phi \right)\mathrm{dV}\right)+\overset{}{\underset{A}{\int }}n\cdot \left(\rho \phi u\right)\mathrm{dA}=\overset{}{\underset{A}{\int }}n\cdot \left(\Gamma \mathrm{grad}\phi \right)\mathrm{dA}+\overset{}{\underset{\mathrm{CV}}{\int }}S_{\phi }\mathrm{dV}$$

The first term of the above formula is the non-stationary term, the second one represents the convective transport of the dependent variable, and the two last ones are the diffusion term and the source term, respectively.

For stationary problems, the first term disappears from Equation (65). However, when considering non-stationary conditions, it is necessary to take into account the change in the value of a dependent variable over time as well. For this purpose, Formula (65) must be integrated over time. The obtained Equation (66) represents the most general form of the transport equation:

(66) $$\overset{}{\underset{\Delta t}{\int }}\frac{\partial }{\partial t}\left(\overset{}{\underset{\mathrm{CV}}{\int }}\left(\rho \phi \right)\mathrm{dV}\right)\mathrm{dt}+\overset{}{\underset{\Delta t}{\int }}\overset{}{\underset{A}{\int }}n\left(\rho \phi u\right)\mathrm{dAdt}=\overset{}{\underset{\Delta t}{\int }}\overset{}{\underset{A}{\int }}n\left(\Gamma \mathrm{grad}\phi \right)\mathrm{dAdt}+\overset{}{\underset{\Delta t}{\int }}\overset{}{\underset{\mathrm{CV}}{\int }}S_{\phi }\mathrm{dVdt}$$

This transformed equation is used in the control volume method for each control cell in the considered computational domain. However, to solve it, the equation must be discretized, i.e., presented in a form that can be solved in each cell into which the area was divided. The equation is then obtained in a discretized form [49]:

(67) $$\frac{\partial \rho \phi }{\partial t}V+\overset{N\;\mathrm{faces}}{\underset{f}{\sum }}\rho_{f}{\;\bar{u}}_{f}\phi_{f}\cdot {\bar{A}}_{f}=\overset{N\;\mathrm{faces}}{\underset{f}{\sum }}\Gamma_{\phi }\nabla \phi_{f}\cdot {\bar{A}}_{f}+S_{\phi }V$$

where the N faces term means the number of walls limiting the control cell, $\phi_{f}$ represents the value of the ϕ variable penetrating through the cell wall (f), $\rho_{f}{\bar{u}}_{f}{\bar{A}}_{f}$ is the mass flow through the cell wall, $\nabla \phi_{f}$ stands for the gradient of the ϕ variable on the f wall, and V is the cell volume. 

In practice, most of the equations describing real phenomena are non-linear, so Equation (67) can still have a non-linear character, expressed, e.g., by the non-linear dependencies. Therefore, Equation (67) is transformed using various approximation methods. The source term is also linearized. As a result of all these operations, a system of linear algebraic equations can finally be obtained:

(68) $$a_{P}\phi_{P}=\underset{\mathrm{nb}}{\sum }a_{\mathrm{nb}}\phi_{\mathrm{nb}}+b$$

The index “nb” means adjacent control cells relative to a cell with a center P, and the variable b refers to the source term, $a_{P}$ and $a_{\mathrm{nb}}$ are linearized coefficients for $\phi_{P}$ and $\phi_{\mathrm{nb}}$. The resulting system of linear equations is solved using the iterative method.

A pressure-based solver was used in fluidized bed modeling. In this approach, the velocity field is obtained by solving the pressure equation (pressure correction) from the continuity and momentum equations. This means that if the correct pressure field is used to calculate the velocity field, then the calculated velocity field will satisfy the continuity equation. This requires the use of an iterative process, wherein each successive iteration values of pressures (and pressure corrections) and velocities are calculated until they meet the continuity equation with the required accuracy. According to the Equation (67), it is necessary to know the value of the dependent variable ϕ on the walls of a control cell. It must be obtained on the basis of knowledge of the variable value in computational nodes, located in the centers of control cells. Interpolation procedures are used for this purpose. 

The second order upwind interpolation scheme was used in the study. In this method, the value of the dependent variable $\phi_{f}$ on the cell’s wall is calculated based on the values in the center of the nearest cell lying in the opposite direction to the fluid movement, according to the following expression [49]:

(69) $$\phi_{f}=\phi_{\mathrm{upstream}}+\nabla \phi_{\mathrm{upstream}}\cdot \bar{r}$$

where the index ‘upstream’ means the closest neighboring cell situated in the opposite direction to the direction of the fluid flow, and $\bar{r}$ stands for the vector connecting the center of that cell with the center point on the wall for which the value of the variable ϕ is calculated.

In the case of a regular grid, it can be written as follows:

(70) $$\phi_{e}=\phi_{P}+\nabla \phi_{P}\cdot \frac{1}{2}\Delta x$$

which will lead to the following expression:

(71) $$\phi_{e}≅\frac{3}{2}\phi_{P}-\frac{1}{2}\phi_{W}$$

### 3.6. Interphase Interactions, Particle–Particle Interaction, and Bed Material Recirculation

It has to be noted that in the considered case of the reactor, enough particles are present such that momentum exchange between dispersed and carrier phase interfaces alters the dynamics of the carrier phase. Due to this fact, it was obviously required to pay attention to the interphase effects that are especially important from the point of view of the turbulence modeling. 

The source term F for the momentum exchange due to particle flow through the control volume has been calculated based on the general rule expressed by Equation (72) below [49]:

(72) $$F=\sum^{\;}\left(\frac{18\mu C_{D}Re}{\rho_{p}d_{p}^{2}24}\left(u_{p}-u\right)+F_{pther}\right){\dot{m}}_{p}\Delta t$$

where: μ—viscosity of the fluid phase material, $\rho_{p}—$the density of the particle, $d_{p}—$the diameter of the particle, Re—Reynolds number, $u_{p}—$the velocity of the solid phase particle, u—velocity of the fluid element, $C_{D}—$drag coefficient, ${\dot{m}}_{p}—$the mass flow rate of the particles, Δt—time step, and $F_{other}—$other interaction forces.

The numerical model that has been used to carry out the computation process allowed us to simulate turbulent multiphase flow based on the principles formulated by Faeth [50] and Amsden [51]. The additional turbulence production in the appropriate continuous phase transport equation in this approach was considered when the particle diameter was higher than 10% of the turbulence length scale.

The heat transfer Q (source or sink) between the continuous phase and solid particles was incorporated into the model by applying the following Equation [49]:

(73) $$Q=\left[\frac{{\bar{m}}_{p}}{m_{p,0}}C_{p}\Delta T_{p}+\frac{\Delta m_{p}}{m_{p,0}}\left(-h_{fg}+h_{pyr}+\int_{T_{ref}}^{T_{p}}C_{p,i}dT\right)\right]{\dot{m}}_{p,0}+Q_{SR}$$

where: ${\bar{m}}_{p}—$the average mass of the particle in the control volume, $m_{p,0}—$the initial mass of the particle, $C_{p}—$heat capacity of the particle, $\Delta T_{p}—$the temperature change of the particle in the control volume, $\Delta m_{p}—$change in the mass of the particle in the control volume, $h_{fg}—$latent heat of volatiles, $h_{pyr}—$the heat of pyrolysis, $C_{p,i}—$heat capacity of the volatiles, $T_{p}—$the temperature of the particle upon exit of the control volume, $T_{ref}—$reference temperature for enthalpy, ${\dot{m}}_{p,0}—$the initial mass flow rate of the particle injection tracked, and $Q_{SR}—$source expressing the thermal effect of surface reaction (combustion).

A modified stochastic collisions approach has been applied to describe particle–particle interactions collisions. The modification assumed the introduction of so-called “parcels” that represent in statistical meaning several individual solid matter particles. Thus one parcel includes many particles that efficiently reduce computation cost. Collisions of the grouped particles have been estimated stochastically based on the O’Rourke algorithm [52]. This approach assumes two kinds of collision effects: coalescence or/and reflection. The probability of each above-mentioned phenomena is calculated by the collision Weber number (*We_c_*) [52]:

(74) $$We_{c}=\frac{\rho U_{rel}^{2}\bar{D}}{\sigma }$$

where: $U_{rel}$—the relative velocity between two parcels, $\bar{D}$—the average diameter of two parcels, and σ—particle Surface stress.

The above-given expression is calculated for each couple of parcels for which collision has been found.

The approach to control solid-phase mass conservation consisted of controlling the total instantaneous mass of inert material in the computational domain.

The experimental data obtained from the real full-scale boiler was applied in the developed CFD model. In order to take into consideration bed material recirculation, the mass flow rate of the solids coming back to the bed was set according to the average mass flow rate declared in the technical specification of the boiler. Such an approach ensured the mass balance of the solid phase in the boiler based on the monitoring of the solid phase total mass in the computational domain. Although this simplification of the system may affect the agreement between conditions in the simulation and real case, such an approach is acceptable due to the goal of the study, concerning the evaluation of the parameter difference for different operation variants. Assessment of the relative change of selected flow variables was still possible and provided the answer regarding final recommendations.

The final confirmation of the correctness of the developed model will be confirmed in the validation chapter. 

## 4. Geometry and Computational Domain Discretization of the Combustion Chamber

**Figure 2:**
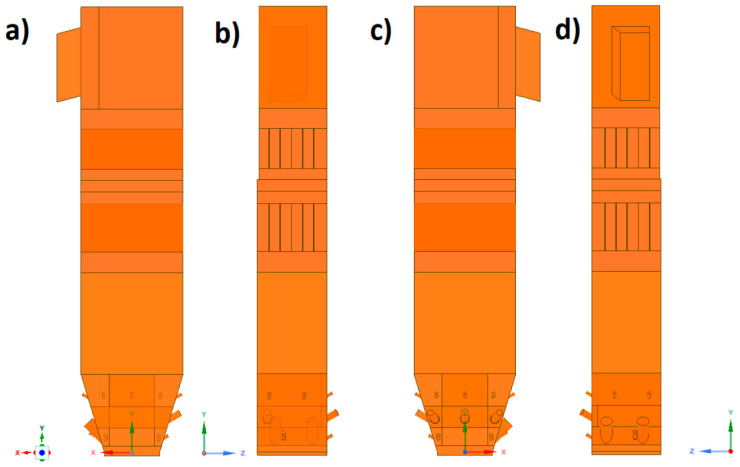
Spatial geometry of the considered part of the boiler: (**a**) view from the side of the symmetry plane, (**b**) view from the front, (**c**) view from the right side, and (**d**) view from the back.

The numerical model based on computational fluid mechanics was performed using the ANSYS Workbench 17.2 platform. The geometric model of the boiler was developed in the ANSYS SpaceClaim Direct Modeler. The real dimensions of the tested part of the installation were applied to build spatial geometry. Due to the presence of the geometric and physicochemical symmetry plane, it was possible to reduce the geometry to half of the considered object. Such an approach allowed studying the system with a reduced number of grid control cells, which had a positive effect on the computation time (Figure 2).

**Figure 3:**
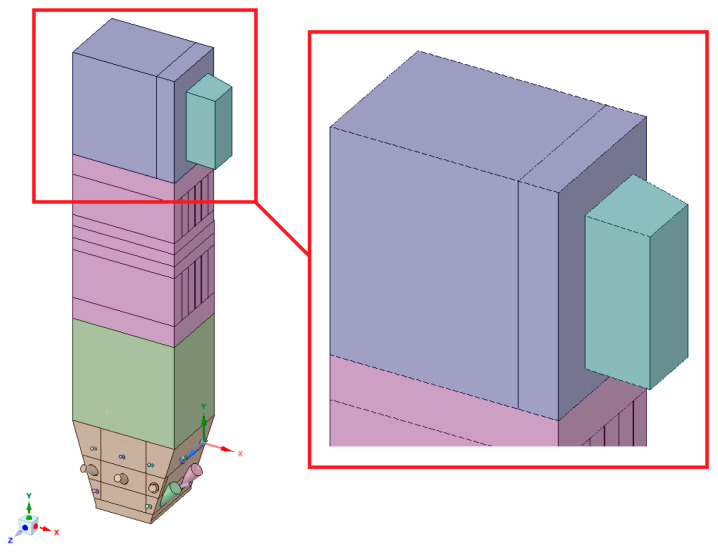
Detailed view of the separated geometry of the exit zone of the riser.

**Figure 4:**
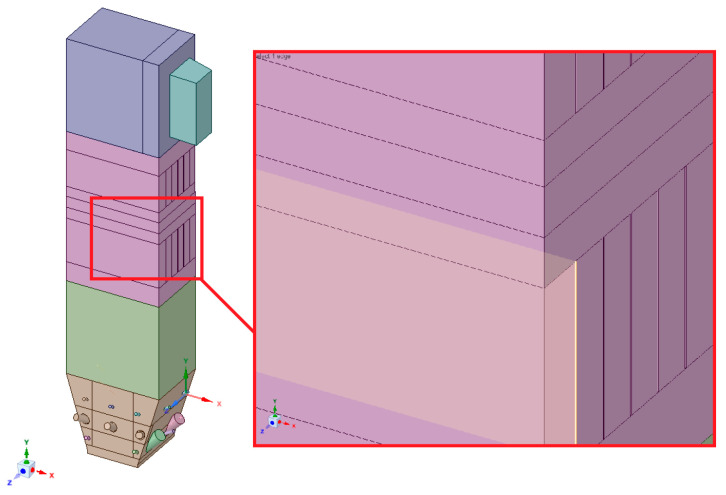
Detailed view of the section between platen superheaters.

**Figure 5:**
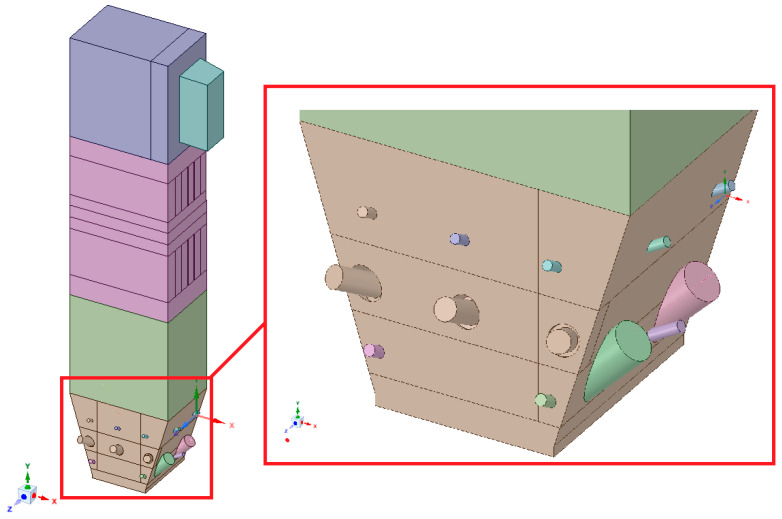
Detailed view of separated sections of the wind box area.

The geometry was divided into orthogonal components (Figure 3, Figure 4 and Figure 5), with the whole domain defined as topologically coherent, which also allowed to generate a coherent computing grid, not requiring the use of so-called computational coupling interfaces of individual parts forming the spatial geometry of the domain.

Figure 4 shows how the zone between the platen superheaters section was divided into three zones. Such an approach enabled buffer passage of grid control cells from smaller volumes in the zone within the exchangers to larger volumes in the area between the superheaters. The division mentioned above also allowed one to reduce the negative impact of narrow passages within the superheater and reheater on the effectiveness of the grid generation algorithm. 

The most complex computing domain was the wind box (Figure 5), having numerous cylindrical outlets for secondary air nozzles, feeders, and burners. Direct contact of arches and all kinds of curves with cuboid parts of the geometry significantly disturbs the grid structure, which may adversely affect the quality of calculations. Therefore, a possibly large number of orthogonal sections were separated from the wind box. In order to avoid deformations of the mesh at the edges of contact of the burners with the plane of the wind box wall, the gas supply pipes were not considered. Such an approach assured that the geometric structure of this part of the domain to be entirely consistent.

After preparing the spatial geometry, the data was exported to the ANSYS meshing module. Preparation for the discretization process started with the task of the required global grid settings shown in Table 4.

The maximum allowed radius of curvature between adjacent tops of grid cells on curves was equal to 18°. Since all sections of spatial geometry were described by local dimensioning of cells (so-called “Body Sizing”), global grid settings in the range of acceptable control cell size were omitted in the considerations.

By setting the “Relevance Center” option (“Fine” mode), maximum quality requirements were imposed on the shape of the grid cells created in the computational domain.

In addition, in order to ensure the highest possible mesh quality with the smallest number of cells, additional local settings were used for subsequent separated sections of spatial geometry. 

**Figure 6:**
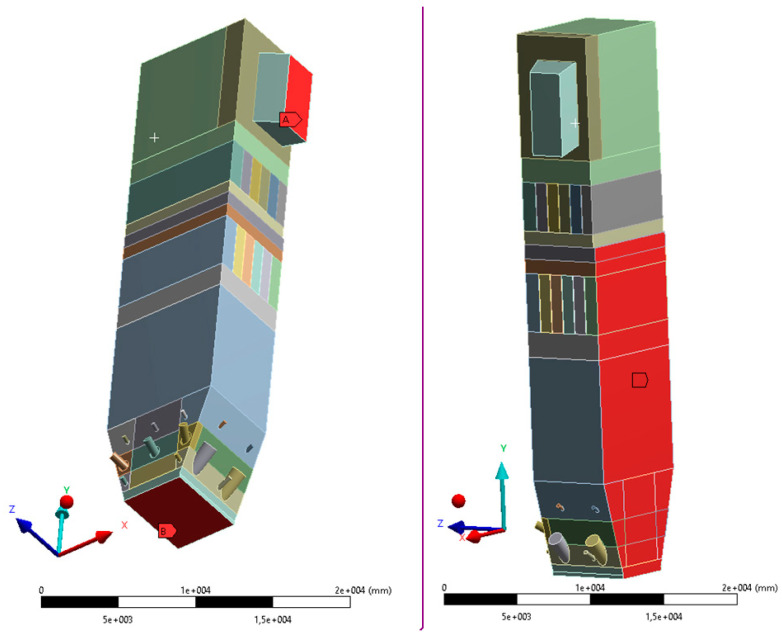
View of selected defined surfaces: left—exits to the cyclone “A” and primary air inlet “B”, right—planes of model symmetry.

**Figure 7:**
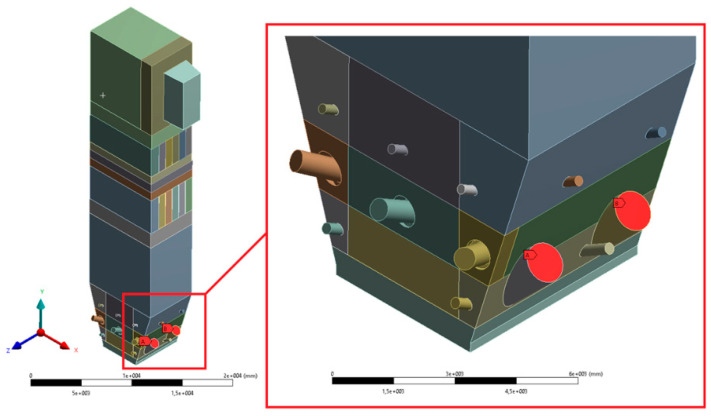
View of selected defined areas—feeders.

**Figure 8:**
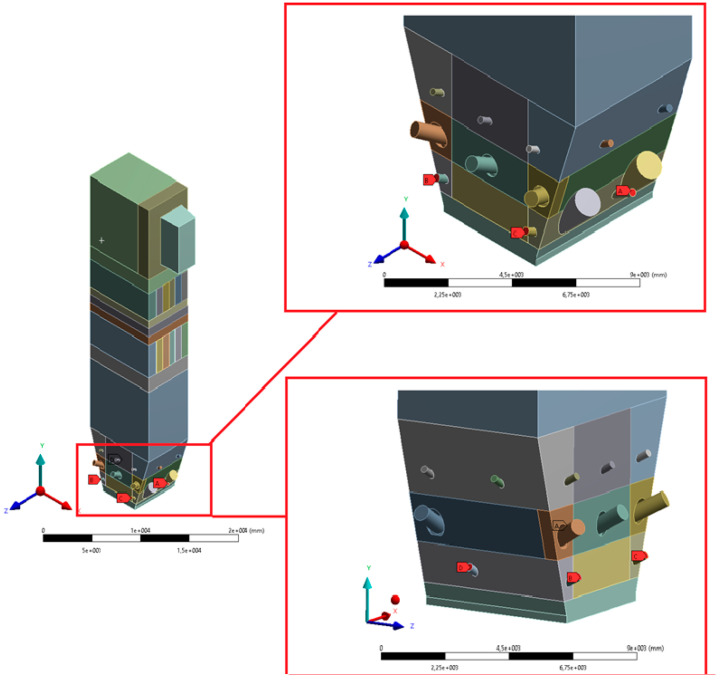
View of selected defined areas—secondary air (I stage) inlets.

**Figure 9:**
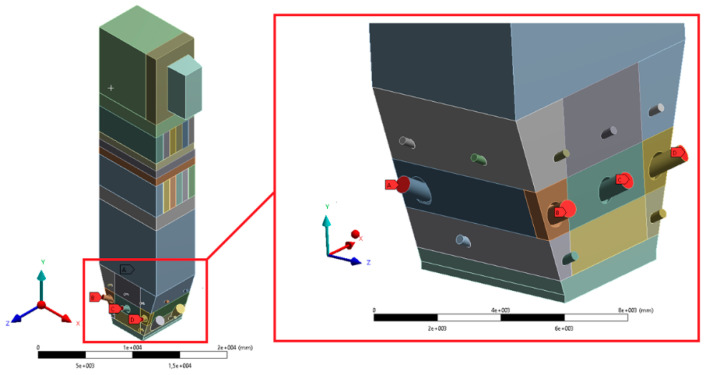
View of selected defined surfaces—start-up burners and secondary air (IInd stage) inlets.

**Figure 10:**
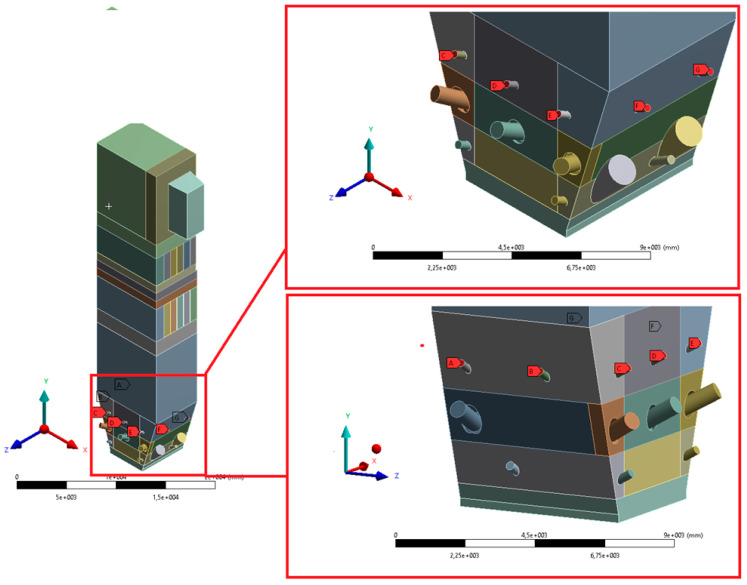
View of selected defined areas—secondary air (IIIrd stage) inlets.

The size of a single grid element (understood as the radius of the sphere described on the element) was between 40 and 60 mm. Such a dense mesh is necessary to model the transport of fluidized bed particles. The defined surfaces, used when working with the Ansys Fluent solver preprocessor, are shown in Figure 6, Figure 7, Figure 8, Figure 9 and Figure 10.

The total number of grid nodes was 21.5 million. The number of mesh elements amounted to 28 million. In order to assess the created grid, the study of critical parameters was carried out, mainly orthogonal quality and skew. Both parameters took values from 0 to 1. In the case of orthogonal quality, the result “1” means the highest possible quality, while the criterion for the grid admission to the calculation is to achieve the result for the worst cell (minimum value of orthogonal quality in the whole grid) not lower than 0.05. In the case of skew, “0” means the highest possible quality, and the grid acceptance criterion is a worst-case cell score of no more than 0.95. 

The grid parameters determined when assessing its quality are listed in Table 5.

**Figure 11:**
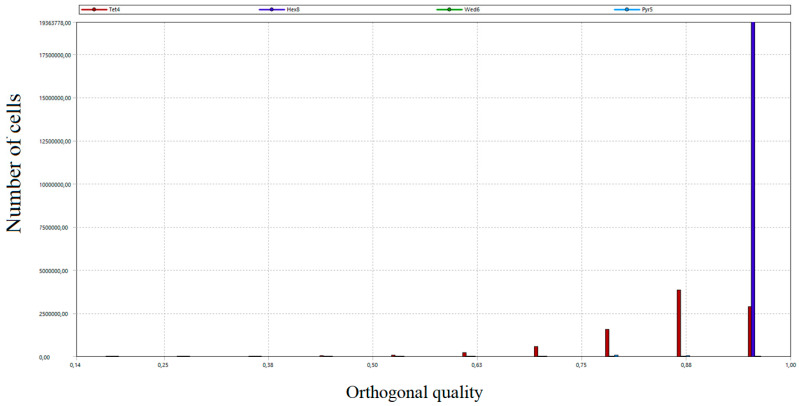
Distribution of the number of cells of the generated grid according to the orthogonal quality.

**Figure 12:**
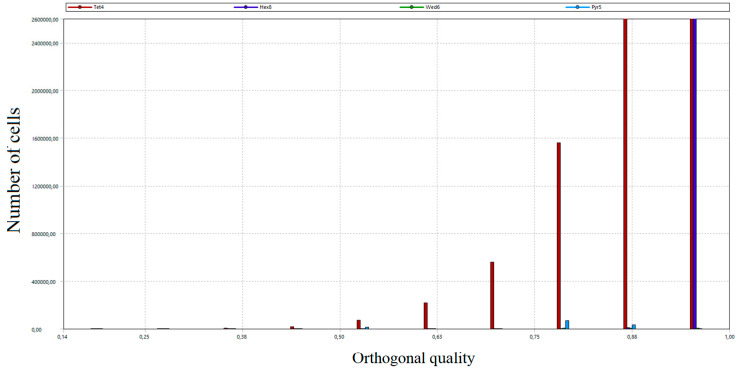
A detailed view of the number of cells distribution of the generated grid according to the orthogonal quality.

**Figure 13:**
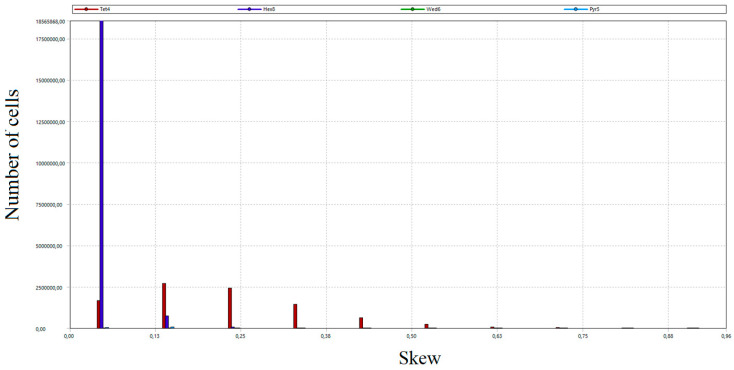
Distribution of the number of elements of the generated calculation grid according to the skew.

**Figure 14:**
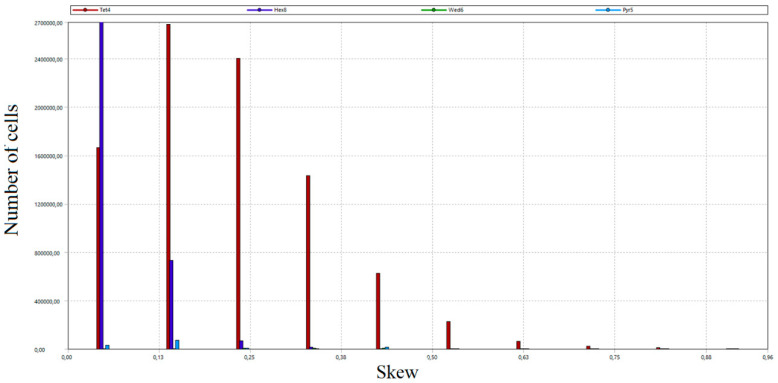
A detailed view of the number of cells distribution of the generated grid according to the skew.

As a part of the grid quality evaluation process, the distribution of the number of grid cells according to orthogonal quality and skew was developed [53]. The results are presented in Figure 11, Figure 12, Figure 13 and Figure 14. Diagrams in Figure 12 and Figure 14 provide detailed views of the respective diagrams where the maximum values of the “*Y*” axis have been reduced to enable an analysis of the distributions in the lower quality range as diagram bars.

According to the diagrams, the grid created was dominated by hexagonal cells with a high degree of orthogonality, characteristic of the highest quality structural grids. Quadrilateral cells can also be distinguished, located primarily in the wind box section, which is too complex in shape to be able to use only cubic elements.

**Figure 15:**
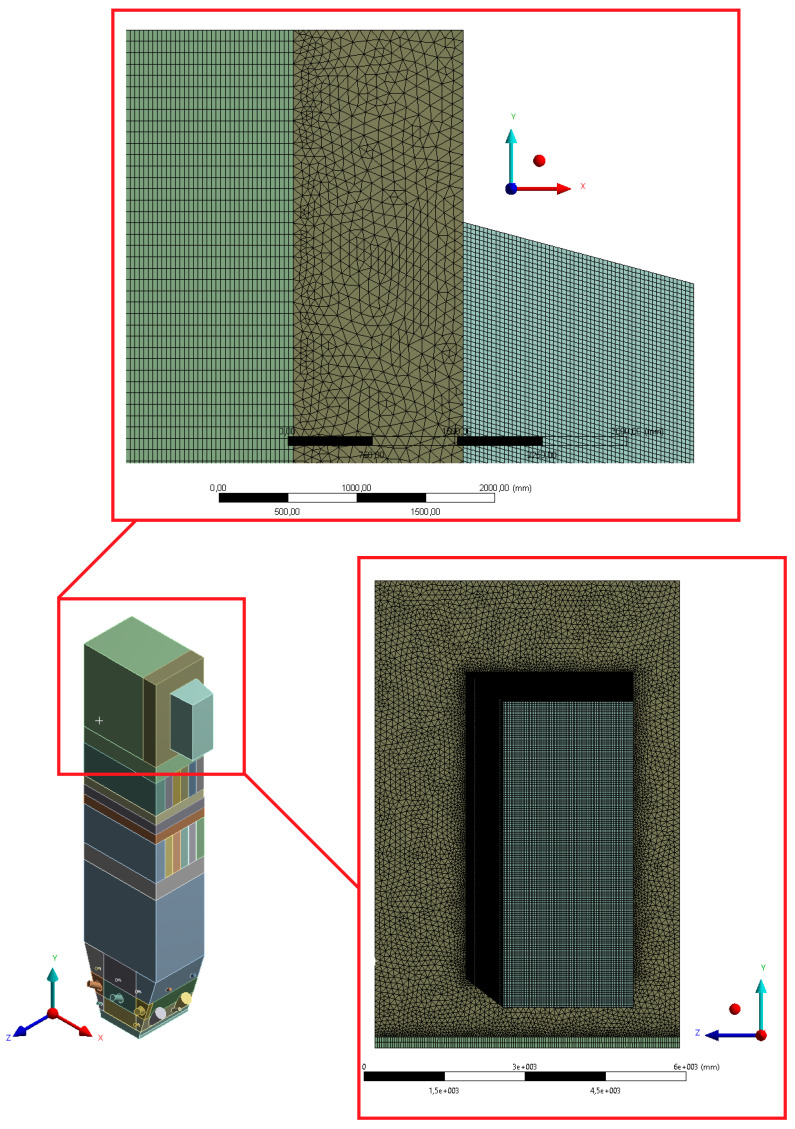
Detailed view of the mesh for the cyclone outlet section.

**Figure 16:**
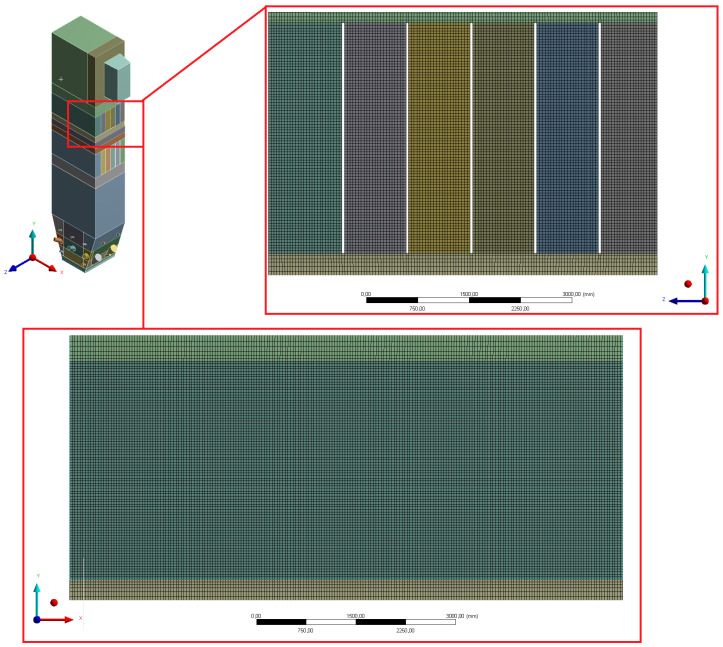
Detailed view of the mesh for sections of platen superheaters.

**Figure 17:**
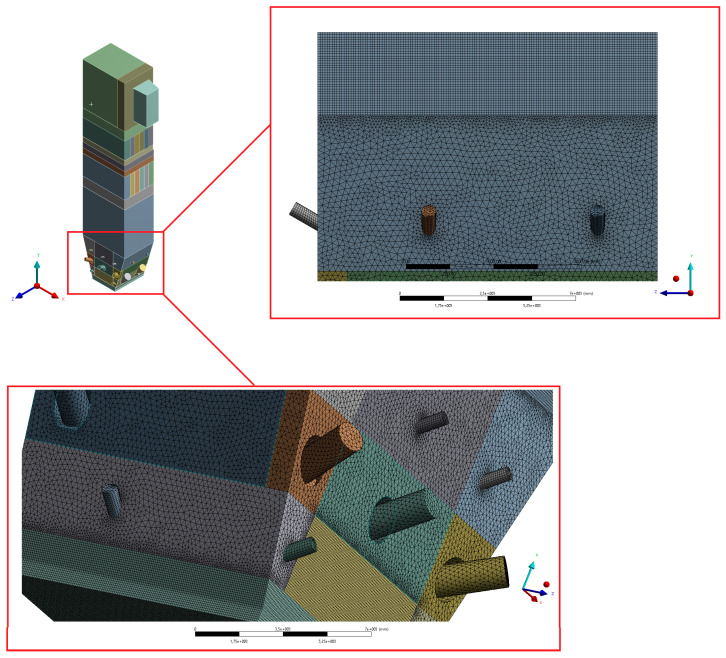
Detailed view of mesh for the wind box section and the area between the wind box and the platen superheater.

The above mentioned tetragonal elements are used in the wind box section, but also between the highest part of the combustion chamber and the domain section reflecting the exit duct to the cyclone. In this case, the use of the tetragonal grid is aimed at creating a kind of buffer layer. It allows the grid to pass from the orthogonal part of the combustion chamber to the diagonal outlet section so that it is possible to use the grid of hexagonal elements at the exit of the computational domain. Such an approach is essential for avoiding possible calculation errors at the outlet (the so-called numerical diffusion). Figure 15, Figure 16 and Figure 17 show selected details of the generated grid.

## 5. Boundary Conditions 

Such geometry performed in the ANSYS meshing module was exported to the ANSYS Fluent solver preprocessor. Before the calculations, all necessary solver settings were defined. The solution to the flow issue was based on the pressure field in the domain (the so-called “Pressure-Based” method), which is recommended for issues concerning relatively low fluid flow velocities. Time-averaged calculations were conducted, which allow significantly reducing the computation time, especially necessary in case of such large and complex cases. The energy equation required for non-isothermal problems was introduced. The k-omega BSL model was applied to the turbulence field solution. A sophisticated model of heat transport through radiation was applied, assuming the effect of the reactive mixture components (carbon dioxide and water) on the radiation (the so-called “Weighted Sum of Gray Gases Model”), based on the concentration of a mixture component.

The discrete phase model (DDPM) was activated to conduct the fluidized bed simulations. Appropriate modifications of transport equations for the reactive mixture (combustion) with reactions were made, taking into account heterogeneous, surface processes (release of volatiles from fuel particles and char burnout) as well as homogeneous processes (combustion in gaseous phase).

Combustion reactions were based on a model assuming close interaction with turbulence in the flow, where the effectiveness of the reaction was dependent on the mixing of reagents (the so-called “Eddy Dissipation Model”).

The following coal properties were taken into account in the calculations: lower heating value 16.7 MJ/kg, volatile 36.6%, ash 20.6%, moisture 21.7, carbon content 85%, hydrogen 10%, oxygen 4%, and nitrogen 1%. 

The first stage of conversion of the coal particles was moisture release:

(75) $$H_{2}O_{Fuel}\overset{\dot{r_{M}}}{\to }H_{2}O_{Vapor}$$

(76) $$\dot{r_{M}}=\{\begin{array}{c} f_{M}\frac{\left(T_{P}-T_{evap}\right)\rho_{M}C_{pm}}{\Delta H_{M}\delta t},\;T_{P}\geq T_{evap}\\ 0,\;T_{P}<T_{evap} \end{array}$$

where: $T_{evap}—$defined evaporation temperature, $\rho_{M}$—the density of the moisture in fuel, $C_{p}$—specific heat of the water in fuel, $\Delta H_{M}$—evaporation heat of the water in fuel, δt—time step, $T_{P}$—the local temperature of the solid fuel, and $f_{M}$—mass fraction of the moisture in the fuel.

The devolatilization rate in Equations (75) and (76) was determined based on the interphase reaction kinetics. It described the process of releasing volatiles from the fuel particles to the gas phase in which homogenous combustion reactions were occurring.

(77) $$Volatiles_{Fuel}\overset{R_{dev}}{\to }Volatiles_{Gas\;phase}$$

(78) $$R_{dev}=A_{dev}e^{\left(\frac{-E_{a,\;dev}}{RT}\right)}\cdot Y_{vol\_fuel}\cdot \rho_{fuel},\;\left[\frac{kg}{m^{3}s}\right]$$

where: $Y_{vol}$—mass fraction of volatiles and $\rho_{pal}$—the density of the solid fuel.

Volatiles were treated as an individual, contractual molecule whose chemical formula was determined based on fuel parameters, and especially it’s composition.

The chemical formula of the volatile molecule can be found on the left-hand side of the expression (79). It was a substrate in only one homogenous reaction in the reference-boiler-state model.

(79) $$C_{1.90}H_{4.69}O_{0.11}N_{0.0337}+3.02O_{2}\to 1.90CO_{2}+2.34H_{2}O+0.0168N_{2}$$

Heterogeneous (surface) combustion of the char remaining after devolatilization was described according to Equations (80) and (81):

(80) $$Char+O_{2}\overset{R_{c}}{\to }CO_{2}$$

(81) $$R_{c}=A_{c}e^{\left(\frac{-E_{a,c}}{RT}\right)}\cdot Y_{c\_solid}\cdot \rho_{pal},\;\left[\frac{kg}{m^{3}s}\right]$$

where $Y_{c\_solid}$—char mass fraction.

The domain material is defined as a reaction mixture characterized by a variable concentration of components: volatile matter released from solid fuel, nitrogen, vapor, carbon dioxide, and oxygen. The following properties of the combustible fuel particle material are determined: density 1600 kg/m^3^, specific heat 1800 J/(kg K), moisture evaporation temperature 400 °C, the heat of the chemical reaction absorbed by the solid phase 30%, and particle diameter 200 μm. 

The following properties of the inert material were applied for calculations: density 2600 kg/m^3^, specific heat 880 J/(kg K), and particle diameter 200 μm. 

The heat transfer between the domain and the combustion chamber walls was considered using convection and radiation models. 

The planar symmetry condition allowed only half of the system to be simulated. According to this, some other parameters related to symmetry were modified (mass flow and hydraulic diameter of primary air).

The boundary and initial conditions were as follows:

- Primary air: mass flow rate 100 kg/s, temperature 243 °C, hydraulic inlet diameter 5.1337 m, and turbulence intensity 10%,
- Four secondary air inlets (I stage): hydraulic diameter 0.285 m, mass flow rate of 2.5 kg/s, temperature 251 °C, and turbulence intensity 5%,
- Four secondary air inlets (II stage, start-up burners): hydraulic diameter 0.56 m, mass flow rate 2.3 kg/s, temperature 251 °C, and turbulence intensity 5%,
- Seven secondary air inlets (III stage): hydraulic diameter 0.245 m, mass flow rate: 1 kg/s, temperature 251 °C, and turbulence intensity 5%,
- Exit from the domain to the cyclone: hydraulic diameter 2.9981 m, pressure 300 Pa, and turbulence intensity 5%,
- Two feeders with a total fuel flow rate of 72 t/h.

The total mass of the fluidized bed is 50 tons. No limestone or any other additions were supplied. 

In order to improve the convergence of the energy equation calculations, the heat transfer through external surfaces of cylindrical elements air nozzles’ outlets outside the wind box was omitted.

Due to the inclusion of gravity in the model and strong mass interactions in the system, a parallel scheme (“Coupled”) was applied. All the governing equations in the model were solved based on the “Upwind-Second Order” spatial discretization scheme.

Calculations were performed for a boiler operating at maximum capacity. After solving the reference issue described by the above settings, a variant analysis was carried out consisting of a gradual replacement of the secondary air streams with syngas (Table 3).

The previously described model has been extended with additional components of the reactive mixture, to consider the new cases with the syngas employment.

The following additional reactions have also been introduced:

(82) $$H_{2}+0.5O_{2}\to H_{2}O$$

(83) $$CO+O_{2}\to CO_{2}$$

(84) $$2CH_{4}+3O_{2}\to 2CO+4H_{2}O$$

All the homogenous gas-phase reactions have been considered as the fast chemistry processes. Reaction rates were calculated based on the Eddy Dissipation approach.

The mass flow rate of the syngas was the same as the previously supplied secondary air mass flow rate. At the same time, the mass flow of solid fuel (coal) in the considered case was reduced by the equivalent of chemical energy supplied to the combustion chamber with the syngas. The decrease in fuel mass in the system was complemented by the same increase in the mass of inert material.

**Figure 18:**
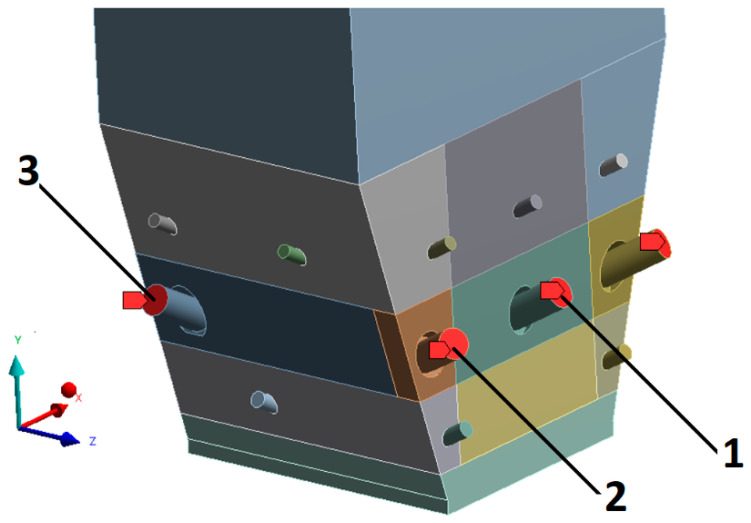
Syngas feeding points in variants K0, K1, K2, and K3.

The following three variants of gas supply to the combustion chamber were analyzed (Figure 18):

1. The use of nozzle no. 1, a total 4.6 kg/s of syngas was supplied through two side start-up burners (variant “K1”),
2. Simultaneous use of nozzles No. 1 and No. 2—a total of 9.2 kg/s of syngas was supplied to the combustion chamber through four side start-up burners (variant “K2”),
3. Simultaneous use of nozzles No. 1, No. 2, and No. 3—a total of 13.8 kg/s of syngas was supplied to the combustion chamber through four side start-up burner and two front burners.

The reference variant, corresponding to the conventional, monocombustion of bituminous coal, is marked with the symbol “K0”.

The syngas mas flow rate in each case was equal to 2.3 kg/s, which corresponds to 14.01 MJ of energy from gas, which corresponds to 0.839 kg/s of the considered coal.

## 6. Validation

**Figure 19:**
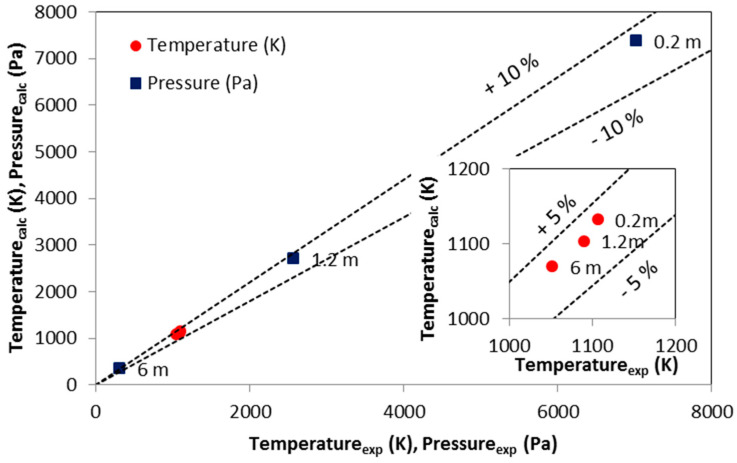
Comparison of pressures and temperatures measured and calculated by the developed CFD model (labels indicate height above the grid).

Based on reports from the boiler thermal measurement, which have been shared with authors by the boiler operator, it was possible to compare data obtained based on the numerical simulation with measurement results. Figure 19 shows the comparison between the measured and calculated pressures and temperatures. 

The developed CFD model was successfully validated against experimental data [54]. The good performance of the developed CFD model was achieved. The maximum relative error was lower than 10% for pressures and 5% for temperatures. Such low relative errors form a solid basis for the possibility of using the developed model in practice. 

The simulation results are described in Part 2 of the paper.

## 7. Conclusions

An interesting idea of multi-fuel combustion in a circulating fluidized bed was considered in the paper. A comprehensive 3D CFD model of bituminous coal and syngas co-combustion in an existing large-scale OFz-425 CFB boiler was proposed. The model considers complex processes that occur in the furnace of the boiler, including dynamics of the fluidized bed, reactions, and heat transfer inside the boiler.

Four different operating scenarios were considered, including the reference variant, corresponding to the conventional, monocombustion of bituminous coal, and three tests involving the replacement of secondary air and part of the coal stream with syngas fed by start-up burners.

The model was successfully validated on the experimental results. The maximum relative error between measured and calculated data was lower than ±10%.

The detailed results of the simulations were described in part II of this paper.

## Figures and Tables

**Table 1:** Technical data of the large-scale OFz-425 CFB boiler.

Boiler Type	OFz-425
Nominal thermal capacity, MW_th_	335.8
Live steam temperature, °C	560
Live steam pressure, MPa	16.1
Secondary steam temperature, °C	560
Secondary steam pressure, MPa	4.0/3.8
Feedwater temperature, °C	250
The efficiency of the boiler, %	91
Maximum continuous rating, t/h	425
Live steam temperature, °C	560
Secondary steam flow rate, t/h	371.2–382.0
Feedwater pressure, MPa	17.9
Excess air in the furnace,	1.2
The temperature of the fluidized bed, °C	850–870
Fluidizing gas velocity, m/s	5–8

**Table 2:** Properties of coal (as received).

Lower heating value (LHV), MJ/kg	16.7
Proximate analysis ^ar^, wt %	
Fixed Carbon ^(by difference)^ FC	21.1
Volatile, V	36.6
Ash, A	20.6
Moisture, W	21.7
Ultimate analysis ^ad^, wt %	
Carbon, C	44.5
Hydrogen, H	3.62
Oxygen, O	12.89
Nitrogen, N	1.17
Sulfur, S	1.8

**Table 3:** Properties of syngas.

Lower heating value (LHV), MJ/kg	6.09
Temperature, °C	128
Pressure, bar	9.6
Mole mass, kg/kmol	21.45
Density, kg/m^3^	6.228
Viscosity, Pas	1.84 × 10^−6^
Specific heat, kJ/(kg K)	1.623
Thermal conductivity, W/(m K)	0.0568
Composition, mol%	
H	24.70
CO	18.84
CO_2_	17.55
COS	0.01
CH_4_	3.39
H_2_O	26.13
H_2_S	0.28
NH_3_	0.15
N	8.44
Ar	0.51

**Table 4:** Global grid settings.

**Defaults**	
Physics Preference	CFD
Solver Preference	Fluent
Relevance	0
Element Midside Nodes	Dropped
**Sizing**	
Size Function	Proximity and Curvature
Relevance Center	Fine
Initial Size Seed	Active Assembly
Transition	Slow
Span Angle Center	Fine
Curvature Normal Angle	Default (18.0°)
Num Cells Across Gap	3
Proximity Size Function Sources	Faces and Edges
Min Size	Default (5.77020 mm)
Proximity Min Size	Default (5.77020 mm)
Max Face Size	100.0 mm
Max Tet Size	Default (1154.0 mm)
Growth Rate	Default (1.20)
Automatic Mesh-Based Defeaturing	On
Defeature Size	Default (2.88510 mm)
Minimum Edge Length	38.0 mm
**Inflation**	
Use Automatic Inflation	None
**Assembly Meshing**	
Method	None
**Advanced**	
Number of CPUs for Parallel Part Meshing	16
Number of Retries	0
Rigid Body Behavior	Dimensionally Reduced
Mesh Morphing	Disabled
Triangle Surface Mesher	Program Controlled
Topology Checking	No
Pinch Tolerance	Default (5.19320 mm)
Generate Pinch on Refresh	No

**Table 5:** Selected parameters of the analyzed grid quality indicators.

**Orthogonal Quality**	
Min	0.14
Max	1.0
Mean	0.95
Standard deviation	8.0779 × 10^−0.02^
**Skew**	
Min	1.3057 × 10^−0.10^
Max	0.95
Mean	75243 × 10^−0.02^
Standard deviation	0.12387

## Nomenclature

ad	air-dried basis
ar	as received
AI	Artificial Intelligence
CFB	Circulating Fluidized Bed
CFBC	Circulating Fluidized Bed Combustor
CFD	Computational Fluid Dynamics
FBC	Fluidized Bed Combustion
WSGGM	Weighted Sum of Gray Gases Model
